# Immunotherapy in head and neck cancer: aiming at EXTREME precision

**DOI:** 10.1186/s12916-017-0879-4

**Published:** 2017-06-02

**Authors:** Petr Szturz, Jan B. Vermorken

**Affiliations:** 1 Department of Internal Medicine, Hematology, and Oncology, University Hospital Brno, Brno, Czech Republic; 2 School of Medicine, Masaryk University, Brno, Czech Republic; 3 Department of Medical Oncology, Antwerp University Hospital, Edegem, Belgium; 4 Faculty of Medicine and Health Sciences, University of Antwerp, Antwerp, Belgium

**Keywords:** Head and neck cancer, Recurrent, Metastatic, Targeted therapy, Immunotherapy, Cetuximab, Nivolumab, Pembrolizumab, Biomarkers, Combination regimen

## Abstract

**Background:**

Locoregionally advanced, recurrent, and metastatic squamous cell carcinomas of the head and neck (SCCHN) remain difficult to treat disease entities, in which systemic treatment often forms an integral part of their management. Immunotherapy is based on functional restoration of the host immune system, helping to counteract various tumour evasion strategies. Broadly, immunotherapeutic approaches encompass tumour-specific antibodies, cancer vaccines, cytokines, adoptive T-cell transfer, and immune-modulating agents. Until 2015, the epidermal growth factor receptor inhibitor cetuximab, a tumour-specific antibody, represented the only Food and Drug Administration (FDA)-approved targeted therapy for SCCHN. Subsequently, in 2016, the results from two prospective trials employing the immune-modulating antibodies nivolumab and pembrolizumab heralded a new era of anticancer treatment.

**Discussion:**

Nivolumab and pembrolizumab are monoclonal antibodies against programmed cell death protein-1 (PD-1), an ‘immune checkpoint’ receptor. Found on the surface of T-cells, PD-1 negatively regulates their activation and can thus be exploited during carcinogenesis. The second-line phase III trial CheckMate-141 randomly assigned 361 patients with recurrent and/or metastatic SCCHN in a 2:1 ratio to receive either single-agent nivolumab (3 mg/kg intravenously every 2 weeks) or standard monotherapy (methotrexate, docetaxel, or cetuximab). Nivolumab improved the objective response rate (13% versus 6%) and median overall survival (OS; 7.5 versus 5.1 months, *p* = 0.01) without increasing toxicity. Exploratory biomarker analyses indicated that patients treated with nivolumab had longer OS than those given standard therapy, regardless of tumour PD-1 ligand (PD-L1) expression or p16 status. In the non-randomised, multicohort phase Ib study KEYNOTE-012, treatment with pembrolizumab achieved comparable results. Importantly, most of the responding patients had a long-lasting response.

**Conclusion:**

Based on recent results, nivolumab and pembrolizumab have been approved by the FDA as new standard-of-care options for the second-line treatment of recurrent and/or metastatic SCCHN. Generally well tolerated, these novel drugs demonstrated modest response rates, with tumour regressions usually being durable, even in platinum-resistant/refractory cases. The next step will be to extend the observed benefit to first-line treatment, currently dominated by the EXTREME regimen (platinum/5-fluorouracil/cetuximab), and to the locoregionally advanced setting, where concurrent chemoradiation with cisplatin is standard. Regimens combining immunotherapy with other modalities will probably further improve outcomes.

## Background

Recently, few topics in oncology have attracted as much attention as immunotherapy. However, marked by several ups and downs, the introduction of immunotherapy into standard anticancer treatment modalities took more than 150 years. In the latter half of the 19^th^ century, the observation of immune infiltrates in neoplastic tissues linked the origin of cancer to sites of chronic inflammation [1]. Independently, experimental attempts with streptococcal culture injections yielded sporadic remissions in patients with inoperable sarcomas [2]. During that period, the Nobel Prize-winner Paul Ehrlich revolutionised our understanding of the role of the immune system in the fight against human diseases by suggesting the existence of specific receptors that are able to bind various antigens. This later evolved into his ‘magic bullets’ theory, which hypothesised the ability to seek out pathogens while sparing healthy tissues. Subsequently, in 1909, he postulated that tumours might be recognised by the immune system [3]. Nevertheless, it was not until the late 1950s that further progress was made. At that time, incorporating emerging discoveries in murine tumour transplantation models and Ehrlich’s conclusions, Thomas and Burnet [4] proposed the concept of immunosurveillance, in which lymphocytes acted as sentinels to protect against transformed cells.

The idea of immunosurveillance was quickly questioned by findings in athymic nude mice exhibiting no increased susceptibility to chemically induced or spontaneous carcinogenesis compared with immunocompetent mice. However, from the 1980s onwards, the prevailing notion began to turn once again as several multifunctional cytokines (e.g. interleukin-2, interferon-α) entered clinical testing, new data on tumour-associated antigens appeared, and adoptive T-cell transfer was used for the first time [4, 5]. Remaining doubts were dispelled in 2001, when Shankaran et al. [6] published their seminal paper showing that deeply immunocompromised mice lacking the recombination activating gene-2 did indeed experience a higher incidence of sarcomas. In the following years, with the arrival of tumour-specific monoclonal antibodies, medical oncology stepped into the era of targeted therapy, expanding the broad spectrum of immunotherapeutic approaches. In addition, as reported in 2010 [7], sipuleucel-T, a vaccine based on autologous dendritic cells, reduced the risk of death in metastatic castration-resistant prostate cancer and became the first therapeutic cancer vaccine to be approved by the United States Food and Drug Administration (FDA) [5].

In parallel, allogeneic bone marrow transplantation, reported for the first time in 1957 [8], also overcame several hurdles to become established as a standard treatment option for selected haematological malignancies. The underlying immune mechanism behind this highly effective form of adoptive T-cell transfer has been referred to as the graft-versus-tumour effect; the ability for engrafted donor lymphocytes to eliminate residual malignant populations in the host organism. The favourable impact of this phenomenon on long-term disease control even reduced the intensity of conditioning chemotherapy and/or irradiation, decreasing patients’ morbidity and mortality [9].

Considering these practice-changing advances in oncology and immunotherapy, a question remained as to whether monoclonal antibodies could effectively target not only malignant cells but also non-cancerous, immunocompetent elements. In the latter setting, the proof-of-principle was provided in 2010, when a large randomised study in patients with metastatic melanoma [10] demonstrated that treatment with ipilimumab, a cytotoxic T-lymphocyte antigen-4 (CTLA-4) blocker, improved overall survival (OS) by 3.5 months compared with a glycoprotein 100 peptide vaccine. Therefore, the current armamentarium of immunotherapeutic strategies includes tumour-specific monoclonal antibodies, cancer vaccines, cytokines, adoptive T-cell transfer, and immune-modulating agents, the latter of which was voted Science magazine’s 2013 ‘breakthrough of the year’ among all scientific disciplines [11].

Head and neck cancers are heterogeneous diseases. Most, arising from the mucosal lining of the oral cavity, larynx, oropharynx and hypopharynx, histologically correspond to squamous cell carcinomas. More than half of patients present with advanced tumours typically requiring a multidisciplinary approach [12]. Single-modality surgery or radiotherapy leads to high cure rates in early disease (stages I and II), but locoregionally advanced squamous cell carcinoma of the head and neck (LA-SCCHN) usually recurs even after aggressive management combining locoregional intervention with systemic therapy. Such cases, being either primary refractory or persistent during primary therapy, or showing locoregional recurrence or distant metastases after an initial response, have poor prognoses [13].

Of the advances made after the introduction of various surgical techniques up until 2015, the following were unequivocally associated with a significant survival benefit: radiotherapy, high-dose three-weekly cisplatin given concurrently with radiotherapy, and cetuximab, an anti-epidermal growth factor receptor (EGFR) monoclonal antibody [14–16]. Cetuximab improved OS in patients with LA-SCCHN treated with definitive radiotherapy and those with recurrent and/or metastatic (R/M)-SCCHN receiving chemotherapy [17, 18]. Preferably, cetuximab should not be prioritised over the standard cisplatin-based chemotherapy when combined with radiotherapy in LA-SCCHN, but it has currently no real competition in first-line palliative systemic treatment [19]. In this respect, the large randomised EXTREME (Erbitux in first-line treatment of recurrent or metastatic head and neck cancer) trial [18] demonstrated that cetuximab could prolong median OS when added to the platinum/5-fluorouracil doublet in R/M-SCCHN (from 7.4 to 10.1 months, *p* = 0.04). Interestingly, no other EGFR-blocking agent has matched these results [13].

One plausible explanation for the success of precision medicine, as seen in EXTREME, is that cetuximab has additional immune-based mechanisms of activity. These stimulate antibody-dependent cellular cytotoxicity (ADCC) and enhance cytotoxic T-lymphocyte cross-priming by dendritic cells [20, 21]. Apart from cell line and mouse models, the importance of ADCC was recently corroborated in patients with LA-SCCHN. In a retrospective analysis [22], high baseline ADCC predicted OS in patients who received radiation concurrently with cetuximab (*n* = 28), but not in patients treated with cisplatin (*n* = 15). In the bioradiation-treated group, patients with high baselines of ADCC (lactate dehydrogenase release, Cytotoxic 96® cytotoxicity assay) and EGFR 3+ (immunohistochemistry) had significantly more complete responses and longer OS than the others.

How can we further leverage the immune system in SCCHN and aim, once again, for such EXTREME precision? The response came early in 2016, when the CheckMate-141 study [23] on nivolumab, an immune-modulating antibody against programmed cell death protein-1 (PD-1), demonstrated an unprecedented survival gain in pre-treated patients with R/M-SCCHN. We summarise current evidence in novel immunotherapeutic approaches in head and neck cancer and outline future avenues for development in this rapidly evolving field.

### Immune dysfunction and restoration

An established hallmark of the multistep evolution of cancer is its ability to avoid immune destruction, particularly by T-lymphocytes and B-lymphocytes, macrophages, and natural killer cells [24]. Immune evasion is often perceived as a failure of immunosurveillance, but this does not fully explain the complex interplay between cancer and immunity. Immunosurveillance represents just part of a broader, dynamic process known as cancer immunoediting, comprising three phases: tumour elimination, equilibrium, and escape to clinically overt disease. Thus, the immune system is capable of both preventing and promoting the formation and growth of neoplastic tissue [4]. Consequently, cancer immunotherapy is based on functional restoration of certain signalling cascades of the host immune system. These cascades help to counteract various tumour evasion strategies such as reduced antigen processing and presentation, increased tumour-permissive cytokine profiles, establishment of an immunosuppressive microenvironment, cellular immune escape via regulatory T-cells or myeloid-derived suppressor cells (MDSCs), and induction of anergic T-cells either by an increase of co-inhibitory receptors (e.g. CTLA-4 or PD-1) or decreased co-stimulatory receptors [25, 26].

The most clinically investigated, co-inhibitory pathways, dubbed ‘immune checkpoints’, regulate the duration and extent of immune system activity, delivering negative signals to prevent autoimmune reactions. As a receptor expressed on CD4^+^, CD8^+^, and regulatory T-cells, CTLA-4 competitively disrupts the axis between tumour-specific T-lymphocytes bearing CD28 receptors and stimulatory ligands CD80 (B7) and CD86 (B70) on antigen-presenting cells. Similarly, PD-1 is a receptor exposed on the surface of activated T-lymphocytes and B-lymphocytes and myeloid elements. The ligands PD-L1 (CD274/B7-H1) and PD-L2 (CD273/B7-DC) are transmembrane proteins found on both normal and cancerous cells, transmitting inhibitory signals that downregulate T-lymphocyte activation. Impaired immune recognition may thus occur when a high fraction of CTLA-4 or PD-1 positive T-cells are found in the tumour microenvironment, or when the tumour itself expresses increased PD-L1 or PD-L2 [26].

Head and neck cancers are an immunosuppressive group of diseases that employ different immune evasion mechanisms. Immune dysfunction has been implicated in carcinogenesis of human papillomavirus (HPV)-positive oropharyngeal cancer as well as most remaining SCCHN cases linked to alcohol and tobacco [26, 27]. The receptor-ligand interplay between PD-1 and PD-L1 is particularly noteworthy. Badoual et al. [28] attempted to explain the markedly better prognosis of HPV-positive tumours of the oropharynx compared with other SCCHN types by examining PD-1 and PD-L1 expression in 64 SCCHN cases, mostly of oropharyngeal origin. Viral positivity was significantly associated with infiltration of PD-1^+^CD4^+^ T-cells (*p* = 0.045) and both PD-1^+^CD4^+^ and PD-1^+^CD8^+^ T-cells (*p* = 0.045), but not PD-L1 status. Infiltration of PD-1^+^ T-lymphocytes was also a favourable prognostic factor in HPV-related disease. As confirmed by others, expression of PD-L1 in tumours is common and detected regardless of HPV status. Pooling data from four studies on head and neck cancer including the nasopharynx, Lin et al. [29] calculated its prevalence as 54%. These results should be interpreted cautiously because of the variable quality in archived tissue specimens, and differences in the assays, scoring methods, and thresholds for positivity used.

Based on gene expression profiling and HPV status, two HPV-positive (mesenchymal, classical) and three HPV-negative (basal, mesenchymal, classical) subtypes were recently identified to overcome some limitations associated with traditional anatomic site and stage-based classification [30]. A key translationally relevant discovery was that both HPV-positive and HPV-negative mesenchymal subtypes demonstrated a prominent immune phenotype with marked CD8^+^ lymphocyte infiltration. Such strong activation of the immune system provides a further rationale for immunotherapy and could become a predictive biomarker for this therapeutic approach. Moreover, in line with the independent observations by Badoual et al. [28], the HPV-positive mesenchymal subtype was associated with a trend towards better OS compared with the HPV-positive classical subtype [30].

### The year of immunotherapy in head and neck cancer: 2016

Multiple preliminary reports have shown immune checkpoint inhibitors to have promising activity in SCCHN. However, until recently, their impact on OS remained unknown. At the annual meeting of the American Association for Cancer Research in April 2016, investigators on the randomised global phase III trial ‘CheckMate-141’ (NCT02105636) [31] declared nivolumab to be the first drug to improve survival in patients with platinum-refractory R/M-SCCHN. As published later [23], the study evaluated the efficacy and safety of nivolumab at an intravenous dose of 3 mg/kg every 2 weeks, versus weekly intravenous single-agent chemotherapy (methotrexate 40–60 mg/m^2^, docetaxel 30–40 mg/m^2^) or cetuximab (400 mg/m^2^ once, then 250 mg/m^2^). Key eligibility criteria were as follows: R/M-SCCHN of the oral cavity, pharynx, or larynx not amenable to curative therapy, disease progression within 6 months after platinum-based chemotherapy given irrespective of clinical setting, good Eastern Cooperative Oncology Group (ECOG) performance status (0 or 1), and no active brain metastases, autoimmune disease, systemic immunosuppression, or previous therapy targeting immune-checkpoint or T-cell co-stimulation pathways. Receipt of prior cetuximab treatment served as a stratification factor. OS was the primary objective, and secondary objectives assessed progression-free survival (PFS) and overall response rate.

Patients enrolled between June 2014 and August 2015 were randomised in a 2:1 ratio to receive either nivolumab (236 of 240 assigned) or a single-agent of the investigator’s choice (111 of 121 assigned). In the intention-to-treat population (*n* = 361), median age was 60 years with 113 (31%) patients being ‘elderly’ (aged 65 or over). The standard therapy arm included a higher percentage of elderly patients, as well as never-smokers, while other characteristics were equally balanced. Fifty-five percent of patients had previously received two or more lines of systemic treatment. Median time on therapy was 1.9 months in each cohort. At data cut-off, 41 of 236 patients (17%) continued treatment with nivolumab as opposed to 3 of 111 (3%) receiving single-agent chemotherapy or cetuximab. Treatment-related adverse events occurred at similar rates in the two arms (59% with nivolumab versus 78%), but grade 3–4 toxicities were less frequent with the experimental drug (13%) than the drug of the investigator’s choice (35%). In the nivolumab-treated group, fatigue (14%), nausea (9%), rash (8%), decreased appetite (7%), pruritus (7%), and diarrhoea (7%) were the most common side effects of any grade, while other toxicities did not exceed 6%. Apart from skin reactions, adverse events with a potential immunologic aetiology comprised endocrine (8%, primarily hypothyroidism), gastrointestinal, hepatic, pulmonary, infusion-related, and renal toxicities. There were two treatment-related deaths in the nivolumab cohort (caused by pulmonary embolism and hypercalcemia) and one in the standard therapy arm (lung infection) [23].

After a median follow-up duration of 5.1 months, subjects assigned to the nivolumab group had a 30% reduction in risk of death compared to the control arm (hazard ratio, 0.70; 97.73% CI, 0.51–0.96; *p* = 0.01). Median OS was 7.5 months versus 5.1 months in favour of nivolumab. At 12 months, OS among patients on nivolumab was more than double that of patients treated with the investigator’s therapy of choice (36% versus 17%, respectively). Correspondingly, immunotherapy induced more objective responses (6 complete, 26 partial, overall rate 13%, versus 1 complete, 6 partial, overall rate 6%), but no differences in median PFS were observed (about 2 months in both groups). Exploratory biomarker analyses suggested that the beneficial survival effect in favour of nivolumab was present regardless of tumour PD-L1 expression or p16 status (both assessed by immunochemistry). Among 260 evaluable patients, PD-L1 membrane staining was detected in at least 1% of tumour cells in 57% of cases. About the same proportion (92 of 178, 52%) tested positive for p16 as a surrogate marker of HPV infection. Pre-specified analyses implied that OS might have been greater for patients treated with nivolumab whose tumours expressed PD-L1 and/or p16, but the interactions were not statistically significant [23].

In 2016, results from another prospective trial of an immune checkpoint inhibitor were published. The non-randomised, multicohort phase Ib trial ‘KEYNOTE-012’ (NCT01848834) [32] recruited patients diagnosed with SCCHN, bladder, triple-negative breast, and gastric cancers. Cohort B consisted of 60 cases of R/M-SCCHN, with or without previous systemic therapy and expressing PD-L1 at a level of at least 1%. Using a schedule of 10 mg/kg pembrolizumab, again an anti-PD-1 antibody, administered intravenously every 2 weeks, the investigators demonstrated efficacy and toxicity outcomes similar to nivolumab in the CheckMate-141 study. The reported overall response rate reached up to 18% (8/45) with a median PFS of 2 months and a 17% (10/60) rate of grade 3–4 drug-related adverse events. Objective responses were also more common in HPV-positive than in HPV-negative patients. Besides that, in the intention-to-treat population (*n* = 61), median OS was 13 months with 51% of patients alive at 12 months, and no deaths were attributed to pembrolizumab. In an expanded KEYNOTE-012 study [33], a B2 cohort of 132 patients with R/M-SCCHN and any PD-L1 expression, HPV status, or prior systemic therapy received pembrolizumab at a fixed intravenous dose of 200 mg every 3 weeks. Some of the observed outcomes here were in line with the CheckMate-141 trial, since 18% (24/132) of the study population experienced an objective response favouring those with PD-L1-positive and/or HPV-positive tumours, median PFS and OS times were 2 and 8 months, respectively, and grade 3–4 treatment-related side effects occurred in 9% of enrolled patients [33]. Altogether, both KEYNOTE-012 cohorts [32, 33] contained a substantial proportion of heavily pre-treated participants, median age ranged between 60 and 63 years, and most of the responding patients had an ongoing response at the time of data cut-off.

How do such results compare to those obtained in randomised studies with other targeted drugs? Table 1 summarises evidence from eight large phase III trials conducted in the R/M disease setting [18, 23, 34–39]. To date, only two molecularly targeted approaches have delivered significantly longer OS than their respective control arms, i.e. cetuximab as an adjunct to the platinum/5-fluorouracil combination in the first-line EXTREME trial, and nivolumab monotherapy in the second-line CheckMate-141 trial. Looking at Table 1 more broadly, results in both first-line and second-line treatments are somewhat homogeneous. However, some classical outcome measures, such as median PFS and OS, or the respective landmark analyses, may not fully capture the exceptional activity of immune-modulating agents. Unlike other targeted drugs (e.g. EGFR-inhibitors) and cytotoxic chemotherapy, checkpoint inhibitors can elicit delayed clinical effects and may also lead to long-term off-treatment survival [40, 41].

**Table 1:** Peer-reviewed data from large phase III trials conducted in patients with recurrent and/or metastatic squamous cell carcinoma of the head and neck

Study, line (year)	*N*	Regimen (treatment arms A, B, C)	Response rate (%)	Median progression-free survival (months)	Median overall survival (months)
ECOG 5397 1^st^ line (2005) [34]	112/	A: P + cetuximab^b^	26^e^	4.2	9.2
	117	B: P + placebo	10	2.7	8.0
EXTREME 1^st^ line (2008) [18]	442/	A: PF/CF + cetuximab^b^	36^e^	5.6^e^	10.1^e^
	442^a^	B: PF/CF	20	3.3	7.4
SPECTRUM 1^st^ line (2013) [35]	657/	A: PF + panitumumab^b^	36^e^	5.8^e^	11.1
	657^a^	B: PF	25	4.6	9.0
IMEX 2^nd^ line (2009) [36]	456/	A: Gefitinib (250 mg)^b^	2.7	ND	5.6
	486^a^	B: Gefitinib (500 mg)^b^	7.6	ND	6.0
		C: MTX	3.9	ND	6.7
ZALUTE 2^nd^ line (2011) [37]	286/	A: Z^b^ + BSC	6.3	2.3^e^	6.7
	286^a^	B: BSC (optional MTX)	1.1	1.9	5.2
ECOG 1302 2^nd^ line (2013) [38]	177/	A: D + gefitinib^b^	12.5	3.5 (TTP)	7.3
	239^a^	B: D + placebo	6.2	2.1 (TTP)	6.0
LUX-Head&Neck1 2^nd^ line (2015) [39]	483/	A: Afatinib^c^	10.2	2.6^e^	6.8
	483^a^	B: MTX	5.6	1.7	6.0
CheckMate-141 2^nd^ line (2016) [23]	361/	A: Nivolumab^d^	13.3^e^	2.0	7.5^e^
	361^a^	B: MTX or D or cetuximab^b^	5.8	2.3	5.1

*N* number of patients analysed for response/efficacy, *P* cisplatin, *C* carboplatin, *F* 5-fluorouracil, *MTX* methotrexate, *Z* zalutumumab, *BSC* best supportive care, *D* docetaxel, *ND* no data, *TTP* time to progression

^a^intention-to-treat population

^b^epidermal growth factor receptor inhibitor

^c^irreversible HER family receptor blocker

^d^programmed cell death protein-1 inhibitor

^e^significant differences

Kaplan–Meier plots typically show a late separation of survival curves in the order of several months with a plateau phase after more than a year, which has important implications for statistics [40, 42]. The biological background of this peculiar manifestation of clinical benefit probably dwells in the time necessary to unlock the natural anticancer potential of the immune system and translate it into a survival effect [40]. In this regard, compared with classical cytotoxic therapies, the proportion of patients with stable disease treated with nivolumab and pembrolizumab halves to about 20%. This behaviour further illustrates the characteristic mechanism of action of this novel drug class and suggests that the greatest benefit might be seen in those achieving an objective response [43].

Pseudoprogression can be observed in about 10% of advanced melanoma patients soon after treatment onset. Although it resembles true neoplastic growth, pseudoprogression merely reflects a transient infiltration of immune cells. This phenomenon is rare in SCCHN, and the possibility of its occurrence should always be weighed against the risk of futile complications during continued immunotherapy beyond tumour progression, and of missed opportunities for switching treatments in a timely manner.

Usually, when assessing response to treatment, both clinical and radiological aspects must be taken into account. This holds especially true for immunotherapy, where deterioration of general status accompanying ambiguous radiological findings indicates disease progression. Alternatively, in cases of sustained clinical benefit, imaging studies revealing tumour size increment should not automatically trigger a change in management, as was already implemented e.g. in the Checkmate 141 protocol, allowing treatment beyond progression [43]. To correctly interpret such atypical radiographic response patterns, specific immune-related response criteria (irRC) were introduced based on data obtained from phase II trials evaluating ipilimumab in advanced melanoma. Contrary to the conventional Response Evaluation Criteria in Solid Tumours (RECIST), the definition of progression according to the irRC requires confirmation by repeat assessment at least 4 weeks after the first suspicious finding, and identification of new lesions does not exclude an objective response [44, 45].

Taken together, in patients with R/M-SCCHN, the PD-1-directed immune checkpoint inhibitors nivolumab and pembrolizumab are well tolerated novel anticancer agents producing a modest overall response rate of about 15% in second-line treatment, but the induced tumour regression is usually durable, even in platinum-resistant/refractory cases. Consequently, both drugs have gained FDA approval and have become new standard-of-care options for the second-line treatment of R/M-SCCHN.

### What is the next step?

It has been more than 125 years since Dr William Coley demonstrated that an induced streptococcal infection can stimulate anticancer immunity. Despite hurdles, it is now beyond doubt that a properly functioning immune system can effectively kill tumour cells. From this point of view, a sensational event such as spontaneous cancer remission, although rare, is scientifically acceptable. This phenomenon was even reported in a patient with laryngeal carcinoma after a period of prolonged pyrexia [46]. Nonetheless, there remain many unanswered questions about how to augment tumour immunogenicity and select potential responders.

There have been growing efforts to identify suitable targets for immunity stimulation, not only by blocking negative regulatory pathways in effector lymphocytes (i.e. CTLA-4, PD-1/PD-L1) but also by enhancing co-stimulatory signals. Within the latter category, agonistic monoclonal antibodies against OX-40 (MEDI0562) and CD137 (urelumab, utomilumab) or a small molecule toll-like receptor 8 agonist (motolimod) have already entered early clinical testing in SCCHN [47]. The use of various combination regimens is also interesting since both chemotherapy (e.g. oxaliplatin, cyclophosphamide) and radiation can initiate effective antitumour immunity by inducing immunogenic alterations in dying and surviving cancer cells. In the first situation, the so-called ‘immunogenic cell death’ leads to dendritic cell activation, which facilitates the presentation of tumour antigens. Alternatively, surviving cancer cells can undergo ‘immunogenic modulation’, which makes them more susceptible to cytotoxic T-lymphocyte-mediated lysis [47, 48]. Despite its inability to trigger immunogenic cell death, cisplatin as the pivotal cytotoxic agent in SCCHN management exerts stimulatory effects on the immune system. It upregulates major histocompatibility complex class I expression, enhances the lytic activity of effector cells, induces their recruitment and proliferation, and downregulates the immunosuppressive components of the tumour microenvironment, including MDSCs and regulatory T-lymphocytes [49].

Rarely, radiotherapy is associated with the abscopal effect, also known as the radiation-induced bystander effect, in which local treatment leads to a response in distant lesions. In experimental mouse models, Deng et al. [50] noted that irradiation induces increased PD-L1 expression on both tumour and MDSCs, which may promote disease relapse. Subsequently, concomitant administration of anti-PD-L1 synergistically controlled tumour growth, and even mediated abscopal regression of distant lesions. Although the underlying mechanism is not entirely understood, the widely discussed immune origin furnishes innovative opportunities for various immunotherapy combinations [51]. Another approach is cytoreductive surgery, which was hypothesised to aid immunotherapy and endogenous anticancer immunity because of a decrease in the potentially immunosuppressive tumour burden [52].

Immunotherapeutic trials are underway in previously untreated, locoregionally advanced SCCHN, with different priorities identified for patients with HPV-positive and HPV-negative subtypes. In HPV-positive patients, encompassing those with good-prognosis oropharyngeal cancer, treatment strategies aim to take advantage of unique, viral-specific tumour antigens (oncogenes E6 and E7) and effectively integrate immunotherapy along with de-escalated (chemo)radiotherapy protocols [47]. However, retrospective analyses imply that, despite the HPV-related aetiology, reduction of treatment intensity is not appropriate for a subgroup of patients with large primary tumours (T4), extensive nodal disease (N3), or heavy smoking history (≥10 pack-years) [53, 54]. Such cases should be preferably approached in the same way as HPV-negative tumours, which are characterised by a qualitatively different mutational burden and a markedly worse prognosis, thus underscoring the major unmet need to intensify multimodal treatment [47, 55]. Intriguingly, mutational load and a strong neoantigen landscape, both leading to increased immunogenicity, correlate with clinical benefit achieved by checkpoint blockade in other solid tumours [56, 57].

Managing locoregionally advanced disease has been challenging, often involving systemic anticancer agents that are usually administered concurrently with radiotherapy, or sometimes as induction chemotherapy. In nasopharyngeal cancer, adjuvant systemic anticancer agents are given after definitive chemoradiation.

Various immunotherapies are being investigated to improve outcomes in all three of these clinical situations. Table 2 provides an overview of current randomised trials employing these strategies in the curative setting in head and neck cancer. Four studies explore inhibition of the PD-1/PD-L1 axis in combination with definitive radiotherapy with or without cisplatin or cetuximab (NCT02707588, NCT02999087, NCT03040999, and NCT02952586). Two trials focus on adjuvant immunotherapy; in nasopharyngeal carcinoma using autologous tumour-infiltrating lymphocytes (NCT02421640), and in other head and neck cancer subsites using pembrolizumab (NCT02841748). RTOG 3504 examines the efficacy and safety of nivolumab in the definitive and adjuvant settings (NCT02764593). Finally, IRX-2 (citoplurikin), a primary human cell-derived biologic with multiple active cytokine components, is being tested in a randomised phase II trial of neoadjuvant and adjuvant therapy in patients with newly diagnosed curative resectable stages II, III, or IVA oral cavity cancer (NCT02609386).

**Table 2:** Ongoing randomised trials with selected immunotherapeutics^a^ in locoregionally advanced head and neck cancer (also including nasopharyngeal carcinoma) as of April 2017 (≥ 100 patients)

Trial, ClinicalTrials.gov identifier	Phase, setting	Estimated enrolment	Immunotherapeutic approach	Regimen (treatment arms A, B, C)	Primary completion date
NCT02421640^b^	IIR, adjuvant	116	Adoptive T-cell transfer	A: Autologous TILs^e, f^ B: No adjuvant therapy	3/2017
NCT02707588 (PembroRad)	IIR, definitive	114	Anti-PD-1	A: Pembrolizumab^e^ + RT B: Cetuximab + RT	8/2017
NCT02841748 (PATHWay)	IIR, adjuvant	100	Anti-PD-1	A: Pembrolizumab^e^ B: Placebo	8/2018
NCT02609386^c^ (INSPIRE)	IIR, neo-adjuvant and adjuvant	200	Cytokines	A: IRX-2^e^ + CIZO B: CIZO	2/2019
NCT02764593 (RTOG 3504)	I/III, definitive and adjuvant	120	Anti-PD-1	A: Nivolumab^e^ + low-dose cisplatin + RT B: Nivolumab^e^ + high-dose cisplatin + RT C: Nivolumab^e^ + cetuximab + RT D: Nivolumab^e^ + RT	3/2019
NCT02999087 (REACH)	III, definitive^d^	688	Anti-PD-L1	A: Cisplatin + RT B, C: Cetuximab + avelumab^e^ + RT D: Cetuximab + RT	10/2019
NCT03040999 (KEYNOTE-412)	III, definitive^d^	780	Anti-PD-1	A: Pembrolizumab^e^ + cisplatin + RT B: Placebo + cisplatin + RT	3/2021
NCT02952586 (JAVELIN Head and Neck 100)	III, definitive^d^	640	Anti-PD-L1	A: Avelumab^e^ + cisplatin + RT B: Placebo + cisplatin + RT	4/2021

*IIR* phase II randomised, *TILs* tumour-infiltrating lymphocytes, *PD*-*1* programmed cell death protein-1, *PD*-*L1* programmed cell death ligand-1, *RT* radiotherapy, *CIZO* cyclophosphamide, indomethacin, zinc-containing multivitamin, omeprazole

^a^immune-modulating agents, vaccines, and adoptive T-cell transfer

^b^only nasopharyngeal cancer

^c^only oral cavity cancer

^d^including maintenance therapy

^e^immunotherapeutic approach under investigation

^f^after definitive chemoradiotherapy with cisplatin

Besides immune-modulating agents, HPV positivity opens up promising avenues for further immunotherapeutic interventions. Pioneering clinical trials of HPV vaccines began enrolment in the late 1990s. Subsequently, a vaccine for use in young women for the prevention of cervical, vaginal, and vulvar cancers became commercially available in 2006. The indication was later expanded to also cover genital warts and anal cancer prevention in both genders. Importantly, clinical endpoints in the registration trials were limited to premalignant lesions. Their typical progression is well documented in anogenital cancers, but less so in SCCHN, which requires longitudinal studies comparing incidence rates before and after its introduction to estimate the impact of vaccination [58]. These vaccines, composed of L1 major capsid protein, do not elicit therapeutic effects on existing pre-cancerous or cancerous lesions because of the lack of cytolytic T-cell response. Therapeutic HPV vaccines targeting the E6 and E7 oncogenes are still in early clinical development, but preclinical studies have yielded encouraging results. For example, the VGX-3100 DNA vaccine in combination with electroporation has been investigated in cervical cancer and SCCHN.

Another immunotherapeutic HPV-related approach is adoptive T-cell transfer, which utilises in vitro-genetically modified autologous tumour-infiltrating T-lymphocytes and has demonstrated convincing activity, mostly in haematological malignancies. The presence of distinct non-host antigens (E6 and E7) means that HPV-driven tumours are ideal target candidates [59]. In metastatic cervical cancer, a single T-cell infusion produced an overall response rate of 33% with two cases of complete regression [60]. At least five early clinical trials address this issue in patients with SCCHN (results forthcoming) [59]. Finally, similar considerations pertain to those affected by Epstein–Barr virus (EBV)-positive nasopharyngeal carcinoma. In these cases, vaccines and adoptive T-cell transfer have demonstrated biological activity in boosting the anticancer properties of T-cells, but further efforts must be undertaken to improve outcomes [61]. Various combination strategies representing a viable treatment option are currently being tested such as in a phase III trial, in which randomised patients with EBV-positive nasopharyngeal carcinoma receive either a cytotoxic doublet (gemcitabine plus carboplatin), or the same regimen followed by a reinfusion of autologous EBV-specific T-lymphocytes (Table 3).

**Table 3:** Ongoing randomised first-line trials with selected immunotherapeutics^a^ in recurrent and/or metastatic head and neck cancer (also including nasopharyngeal carcinoma) as of April 2017 (≥ 100 patients)

Trial, ClinicalTrials.gov identifier	Phase	Estimated enrolment	Immunotherapeutic approach	Regimen (treatment arms A, B, C)	Primary completion date
NCT01836029 (ACTIVE8)	IIR	175	TLR8 agonist	A: Motolimod^c^ + PFE B: Placebo + PFE	9/2016
NCT02823574 (CheckMate-714)	IIR	315	Anti-PD-1 Anti-CTLA-4	A: Nivolumab^c^ + ipilimumab^c^ B: Nivolumab^c^ + placebo	2/2018
NCT02551159 (KESTREL)	III	760	Anti-PD-L1 Anti-CTLA-4	A: Durvalumab^c^ B: Durvalumab^c^ + tremelimumab^c^ C: PFE	3/2018
NCT02358031 (KEYNOTE-048)	III	825	Anti-PD-1	A: Pembrolizumab^c^ B: Pembrolizumab^c^ + PF C: PFE	3/2018
NCT02578641^b^	III	330	Autologous EBV-specific CTLs	A: CTLs^c^ + gemcitabine + carboplatin B: Gemcitabine + carboplatin	12/2018
NCT02624999	IIR	100	Vaccine	A: AlloVax™ ^c, d^ B: Cisplatin	12/2018
NCT02741570 (CheckMate-651)	III	490	Anti-PD-1 Anti-CTLA-4	A: Nivolumab^c^ + ipilimumab^c^ B: PFE	1/2019

*IIR* phase II randomised, *TLR8* toll-like receptor 8, *PD*-*L1* programmed cell death ligand-1, *CTLA*-*4* cytotoxic T-lymphocyte antigen-4, *PD*-*1* programmed cell death protein-1, *EBV* Epstein–Barr virus, C*TLs* cytotoxic T-lymphocytes, *PFE* platinum/5-fluorouracil/cetuximab regimen according to the EXTREME trial, *PF* platinum/5-fluorouracil chemotherapy

^a^immune-modulating agents, vaccines, and adoptive T-cell transfer

^b^only EBV-positive nasopharyngeal cancer

^c^immunotherapeutic approach under investigation

^d^bioengineered cell allograft combined with chaperone-rich cell lysate

Every responsible medical decision involves accurately selecting those patients who are most likely to derive clinical benefit from a given intervention, yet this approach has been disappointing in oncology. Theoretically, the need for new medicaments would be noticeably lower if we knew how to precisely use those we already have. Despite indisputable recent advances in managing SCCHN, unfortunately the terms ‘immunotherapy’ and ‘personalised medicine’ do not yet fully overlap. In the EXTREME trial, only 3% (6/222) and 1% (2/220) of patients enrolled in the cetuximab and control arms, respectively, were known to still be alive after 5 years [62]. By contrast, extrapolating results obtained in advanced melanoma, recent data from immunotherapy trials conducted in R/M-SCCHN suggest a several-fold increase in long-term survivorship if novel checkpoint inhibitors are administered. Although at this point, longer follow-up is needed to confirm this for the second-line setting (Table 4), further improvements are to be expected with the use of immune-modulating agents in first-line treatment (Table 3) and with the inclusion of predictive biomarkers.

**Table 4:** Ongoing randomised second-line trials with selected immunotherapeutics^a^ in recurrent and/or metastatic head and neck cancer (also including nasopharyngeal carcinoma) as of April 2017 (≥ 100 patients)

Trial, ClinicalTrials.gov identifier	Phase	Estimated enrolment	Immunotherapeutic approach	Regimen (treatment arms A, B, C)	Primary completion date
NCT02105636 (CheckMate-141)	III	361^d^	Anti-PD-1	A: Nivolumab B: SoC	11/2015
NCT02319044 (CONDOR)^b^	IIR	240	Anti-PD-L1 Anti-CTLA-4	A: Durvalumab B: Tremelimumab C: Durvalumab + tremelimumab	9/2016
NCT02252042 (KEYNOTE-040)	III	466	Anti-PD-1	A: Pembrolizumab B: SoC	5/2017
NCT02369874 (EAGLE)	III	720	Anti-PD-L1 Anti-CTLA-4	A: Durvalumab B: Durvalumab + tremelimumab C: SoC	2/2018
NCT02611960^c^ (KEYNOTE-122)	IIR	160	Anti-PD-1	A: Pembrolizumab B: SoC	1/2019

*IIR* phase II randomised, *PD*-*1* programmed cell death protein-1, *PD*-*L1* programmed cell death ligand-1, *CTLA*-*4* cytotoxic T-lymphocyte antigen-4, *SoC* Standard of Care

^a^immune-modulating agents, vaccines, and adoptive T-cell transfer

^b^in patients with PD-L1 negative tumours

^c^only nasopharyngeal cancer

^d^actual and estimated enrolments were 361 and 506, respectively

A National Cancer Institute working group [47] recommended the following five groups of correlative biomarkers for cancer immunotherapy: tumour-related (e.g. interferon-γ gene signature, PD-1/PD-L1 and CTLA-4 expression, T-cell receptor diversity), peripheral blood mononuclear cell-related (e.g. circulating MDSCs and regulatory T-lymphocytes, virus peptide pools in HPV-positive and shared tumour antigen peptide pools in HPV-negative cases), serum-related (e.g. cytokines, growth factors, antibodies), imaging-related (positron emission tomography/computed tomography), and biomarkers from stool samples and oral swabs for future microbiome studies. However, at present, none of these biomarkers have been prospectively validated, so currently they are all strictly limited to clinical research.

There are several phase III studies in which the standard-of-care treatment for patients with R/M disease in the first-line setting – i.e. the EXTREME regimen with platinum/5-fluorouracil plus cetuximab – is being compared with novel immunological approaches (Table 3). Until the eagerly awaited outcomes of these studies are known, the high response (36%) and disease control rates (81%) of EXTREME justifies its continuous use [18]. However, bringing immunotherapy to the fore raises the question, what is the optimal regimen after its failure? Can we expect that, if EXTREME were to be replaced by immune-checkpoint inhibitors, it would still generate meaningful antitumour activity in second-line treatment, or are there other drugs that might work in such a scenario? Similarly, if LA-SCCHN patients were treated with upfront novel immunotherapy – for example together with curative radiotherapy with or without cisplatin or cetuximab – which cytotoxic drugs or targeted agents could effectively be used afterwards? Although evidence from SCCHN trials is currently lacking, we believe that the subgroup of patients, who are resistant to or who relapse after treatment with immune-checkpoint inhibitors, might thrive with further lines of treatment.

Results from several clinical trials, particularly those of cancer vaccines [63–66], suggest that there might be a synergistic effect of immunotherapy and cytotoxic chemotherapy, with unexpectedly favourable responses to such chemotherapy after induction of immunity [67]. Similar findings have emerged with the use of targeted therapies. For example, retained efficacy of binimetinib, a MEK1/2 inhibitor, was observed after prior immunotherapy with immune-checkpoint inhibitors in NRAS-mutant cutaneous melanoma patients [68]. BRAF inhibition also retained its therapeutic potential in BRAF-mutant tumours progressing on anti-PD-1 medication or on a sequential immunotherapy of high-dose interleukin-2 followed by ipilimumab with or without concurrent radiotherapy [69, 70].

Further trials have been initiated to explore different treatment options in those failing immune-checkpoint inhibitors. Early phase clinical research (single group assignment) offers opportunities to receive nivolumab plus interferon-γ (NCT02614456); PBF-509 (adenosine A2a receptor antagonist) alone or as an adjunct to the anti-PD-1 antibody PDR001 (NCT02403193); or pembrolizumab combined with either hypofractionated radiotherapy (NCT02303990), or with vorinostat (NCT02619253), or with enoblituzumab, a humanised monoclonal antibody against cancer stem cells (NCT02475213). Enoblituzumab is also being investigated together with ipilimumab (NCT02381314).

## Conclusions

Harnessing the immune system has shown tremendous potential to become the real ‘magic bullet’ against cancer, yet further learning and mastery of the tools available is required. Mounting clinical and laboratory evidence supports multimodality management as a rational concept to overcome manifold tumour evasion strategies. Predictive biomarkers may improve the cost-effectiveness of anticancer treatment, help avoid unnecessary toxicities caused by futile applications, and contribute to our understanding of the complex network underlying some of the critical immune functions.

## Abbreviations

ADCC: antibody-dependent cellular cytotoxicity
CTLA-4: cytotoxic T-lymphocyte antigen-4
EBV: Epstein–Barr virus
EGFR: epidermal growth factor receptor
EXTREME: Erbitux in first-line treatment of recurrent or metastatic head and neck cancer
FDA: Food and Drug Administration
HPV: human papillomavirus
LA-SCCHN: locoregionally advanced squamous cell carcinoma of the head and neck
MDSC: myeloid-derived suppressor cell
OS: overall survival
PD-1: programmed cell death protein-1
PD-L1: programmed cell death ligand-1
PFS: progression-free survival
R/M: recurrent and/or metastatic
